# Phytochemicals as Anti-Inflammatory Agents in Animal Models of Prevalent Inflammatory Diseases

**DOI:** 10.3390/molecules25245932

**Published:** 2020-12-15

**Authors:** Seong Ah Shin, Byeong Jun Joo, Jun Seob Lee, Gyoungah Ryu, Minjoo Han, Woe Yeon Kim, Hyun Ho Park, Jun Hyuck Lee, Chang Sup Lee

**Affiliations:** 1 College of Pharmacy and Research Institute of Pharmaceutical Sciences, Gyeongsang National University, 501 Jinju-daero, Jinju, Gyeongnam 52828, Korea; shinsaya@gnu.ac.kr (S.A.S.); byeongjun@gnu.ac.kr (B.J.J.); junseoblee@gnu.ac.kr (J.S.L.); rga_97@gnu.ac.kr (G.R.); dlsel79@gnu.ac.kr (M.H.); 2 Division of Applied Life Science (BK21), Plant Molecular Biology and Biotechnology Research Center (PMBBRC), Research Institute of Life Sciences (RILS), Gyeongsang National University, Jinju 52828, Korea; kim1312@gnu.ac.kr; 3 College of Pharmacy, Chung-Ang University, Seoul 06974, Korea; xrayleox@cau.ac.kr; 4 Research Unit of Cryogenic Novel Material, Korea Polar Research Institute, Incheon 21990, Korea; junhyucklee@kopri.re.kr; 5 Department of Polar Sciences, University of Science and Technology, Incheon 21990, Korea

**Keywords:** plant, phytochemical, anti-inflammation, inflammatory disease

## Abstract

Phytochemicals are known to have anti-inflammatory effects in vitro and in vivo, such as in inflammatory disease model systems. Inflammation is an essential immune response to exogenous stimuli such as infection and injury. Although inflammation is a necessary host-defense mechanism, chronic inflammation is associated with the continuous local or systemic release of inflammatory mediators, non-cytokine mediators, such as ROS and NO, and inflammatory cytokines are strongly implicated in the pathogenesis of various inflammatory disorders. Phytochemicals that exhibit anti-inflammatory mechanisms that reduce sustained inflammation could be therapeutic candidates for various inflammatory diseases. These phytochemicals act by modulating several main inflammatory signaling pathways, including NF-κB, MAPKs, STAT, and Nrf-2 signaling. Here, we discuss the characteristics of phytochemicals that possess anti-inflammatory activities in various chronic inflammatory diseases and review the molecular signaling pathways altered by these anti-inflammatory phytochemicals, with a focus on transcription factor pathways. Furthermore, to evaluate the phytochemicals as drug candidates, we translate the effective doses of phytochemicals in mice or rat disease models into the human-relevant equivalent and compare the human-relevant equivalent doses of several phytochemicals with current anti-inflammatory drugs doses used in different types of chronic inflammatory diseases.

## 1. Introduction

Plants can produce a variety of chemical compounds called phytochemicals [1,2], which include primary and secondary metabolites. Primary metabolites are known to contribute to plant growth and metabolism, while secondary metabolites are involved in several functions such as competition, species interaction, and protection from damage/disease [3,4]. Based on biosynthetic origins, phytochemicals can be classified into three major groups, namely, phenolic compounds, terpenoids, and nitrogen-containing alkaloids/sulfur-containing compounds [5]. For millennia, plants have been used as the primary sources of medicines [6]. Phytochemicals with various therapeutic applications have various biological functions, including anti-inflammatory, anti-allergic, anticancer, antibacterial, antiviral, and analgesic functions [7,8]. Furthermore, approximately 25% of drugs used to treat human diseases have been derived from plants [9]. Plants are an excellent source of many new anti-inflammatory drugs that inhibit various inflammatory processes in the immune system [10]. Inflammation is a critical protective response to microbial infection, cell/tissue injury, and irradiation with characteristic features, such as swelling, redness, heat, and pain [11,12]. Chronic inflammatory responses have been known to induce different types of diseases (autoimmune diseases and immune-related diseases), including multiple sclerosis (brain), rheumatoid arthritis (bones), muscular dystrophy (muscle), asthma (lung), inflammatory bowel diseases (gastrointestinal tract), and atherosclerosis (heart) [13]. The inflammatory response involves the release of inflammatory mediators, such as pro-inflammatory cytokines (interleukin (IL)-1, IL-6, and tumor necrosis factor-alpha (TNF-α) and non-cytokine mediators (reactive oxygen species (ROS) and nitric oxide (NO)) [10]. Therefore, the suppression of these inflammatory responses is key to preventing and treating various immune diseases [14]. With the increasing incidence of inflammatory diseases, many treatment strategies are in clinical trials [15]. The major challenge in treating inflammatory diseases is the diversity and complications of the human body’s inflammatory system [16]. Therefore, a detailed understanding of inflammatory processes is key to finding new molecular targets and developing new drugs to manage immune-related diseases [17]. Furthermore, developing novel anti-inflammatory drugs with adequate specificity, minimal side effects***,*** and high efficacy from various sources is essential. To investigate the anti-inflammatory functions of phytochemicals in several animal models, we searched for articles in PubMed with three keywords (phytochemical, chronic inflammatory diseases, and anti-inflammation) and focused on three main prevalent inflammatory diseases (autoimmune diseases, diabetes/obesity, and neurodegenerative diseases). In addition, we limited phytochemicals to those with proven anti-inflammatory effects in in vivo animal models. Here, we discuss the anti-inflammatory effects of phytochemicals in animal models of prevalent inflammatory diseases and review their molecular signaling mechanisms, with a focus on the inflammation-associated transcription factor pathway.

## 2. Anti-Inflammatory Activity of Phytochemicals in Prevalent Inflammatory Diseases

Inflammation against pathological microorganisms is a critical host-defense mechanism; however, consistent inflammatory conditions are involved in the pathogenesis of various chronic inflammatory disorders, including cancer, inflammatory bowel disease, autoimmune diseases, metabolic diseases, neurodegenerative diseases, and vascular diseases [18]. Therefore, preventing continuous inflammatory responses that lead to detrimental effects is essential for resolving inflammation. Although many anti-inflammatory drugs are currently available, their use is restricted owing to side effects following prolonged duration therapy and high cost [19]. In this respect, there is an increasing need for medicinal phytochemicals with anti-inflammatory activity and fewer side effects than synthetic chemical drugs for the therapeutic management of chronic inflammatory diseases [20]. Here, we discuss anti-inflammatory phytochemicals and their mechanisms of action in an in vivo animal model system for different types of chronic inflammatory diseases and summarize them on the basis of structure-based classification [4,5,21]. Additionally, effective doses of these phytochemicals in animal model systems are translated into human-relevant equivalents, compared to current drugs used in each chronic inflammatory disease.

### 2.1. Autoimmune Diseases

Autoimmune disorders are characterized by chronic inflammatory conditions in which the immune system abnormally attacks its body parts. It includes rheumatoid arthritis (RA) and systemic lupus erythematosus (SLE). Effective therapeutic agents for autoimmune diseases are not available, and the currently used anti-inflammatory drugs have many side effects. They are used to reduce inflammation and relieve pain [22]. Several phytochemicals with anti-inflammatory effects have been widely studied as alternatives for autoimmune disorders.

SLE is a systemic inflammatory disease that affects multiple organs. The disease course is difficult to predict; therefore, effective treatment for SLE has not been achieved so far. Long-term administration of immunosuppressive drugs may induce significant side effects in patients with SLE [23]. Recently, a wide range of evidence has shown that phytochemicals can be new therapeutic agents that produce anti-inflammatory effects and attenuate SLE symptoms with fewer adverse effects [24]. Resveratrol ameliorates symptoms such as glomerulonephritis in pristane-induced SLE mice by decreasing Interferon (IFN)-α serum level and T helper (Th) 1 cell percentage [25]. Apigenin suppresses IFN-γ and IL-17 responses in T cells from a lupus-prone mouse model and decreases cyclooxygenase-2 (COX-2) levels in lupus CD4+ T cells, B cells, dendritic cells, and macrophages, resulting in delayed disease progression [26]. Another flavonoid, astilbin, alleviates disease progression in an SLE-prone mouse model by reducing the production of pro-inflammatory cytokines (IFN-γ, IL-17A, IL-1β, TNF-α, and IL-6) in serum and by decreasing the number of activated T and B cells [27]. Treatment with epigallocatechin-3-gallate (EGCG) mitigates disease severity, such as renal impairment in lupus-prone mice by anti-inflammatory effects, including the reduction of nuclear factor kappa B (NF-κB) activation, the expression of NLRP3, IL-1β, and IL-18, and the enhancement of regulatory T (Treg) cell activity [28]. Soy isoflavones, such as daidzin, glycitin, and genistin, ameliorate disease severity in lupus-prone mice by reducing IFN-γ secretion in T cells induced by mitogens [29].

There are different types of drugs to treat SLE, which include NSAIDs (nonsteroidal anti-inflammatory drugs), corticosteroids, immune system suppressing drug, hydroxychloroquine (HDQ) and belimumab [30]. The goal of SLE treatment is to reduce the symptoms and the long term risk, such as comorbidity by disease activity. However, current drugs have well known to have a wide array of adverse effects. Corticosteroids have severe side effects, although they can control the symptoms of serious lupus flare [31]. Oral prednisone for SLE therapy is currently used under 0.5 mg/kg dosage that is important to minimize total dosage to reduce steroid-induced toxicity [30,32]. Immunosuppressive drugs such as methotrexate and mycophenolate mofetil can also have a severe side effect that blocks the immune system’s ability to fight infection [33]. HDQ, an antimalarial drug, has fewer side effects compared to corticosteroids or immunosuppressive drugs. However, taking doses more than 5 mg/kg HDQ is associated with a risk factor for toxicity [32,34]. Belimumab, antibody blocking immune system, has been used in a dose of 10 mg/kg and has been known to have common side effects, including nausea, diarrhea and fever [35]. When the effective doses of phytochemicals in an animal disease model system were translated into human-relevant equivalents, they ranged from a minimum of 1.6 mg/kg to a maximum of 9.7 mg/kg (Table 1). In particular, apigenin, astilbin, and soy isoflavone have shown anti-inflammatory effects in lupus-prone mice at a dose of 1.6 mg/kg based on human equivalent translation that is lower than the effective dose of current drugs (hydroxychloroquine: 5 mg/kg, belimumab: 10 mg/kg) for treatment of SLE. Therefore, in terms of side effects and effective doses, anti-inflammatory phytochemicals with few side effects could be complementary and alternatives medicines against SLE.

RA is a chronic autoimmune disorder that leads to autoimmune inflammation in the connective tissues of the joints and is the most common inflammatory arthritis [36]. The performance of existing conventional drugs does not resolve the disease because progressive joint destruction continues in patients with RA, and the drugs often produce toxic effects [37]. Accumulating experimental evidence shows that phytochemicals can be used as alternative therapeutic agents against RA [38]. Curcumin has anti-arthritic effects, including the reduction of NF-κB expression in synovial tissue and the level of IL-1β and TNF-α in synovial fluid and serum mitigating arthritic symptoms in adjuvant-induced arthritis rat models [39]. Resveratrol has a protective effect in RA rats by decreasing TNF-α, C-reactive protein (CRP), and myeloperoxidase (MPO) and conversely increasing IL-10 in serum [40]. Oral administration of EGCG to RA-induced mice showed anti-inflammatory effects by decreasing inflammatory cytokines (IFN-γ, IL-6, TNF-α, and IL-1β) and altering immune cell populations, with increasing Treg cells in indoleamine 2,3-dioxygenase (IDO)-expressing dendritic cells [41]. Mangiferin attenuates RA severity and reduces IL-1β, TNF-α, IL-6, and receptor activator NF-κB ligand (RANKL) expression in RA-induced mice by suppressing NF-κB and extracellular signal-regulated kinase (ERK)1/2 activation [42]. P-coumaric acid shows anti-inflammatory effects by decreasing TNF-α in the synovial tissues of RA-induced rats [43]. In an in vivo collagen-induced RA model, genistein exhibits inhibitory effects against inflammation by decreasing IFN-γ secretion and, conversely, increasing IL-4 secretion from spleen lymphocytes [44]. Dietary β-cryptoxanthin, a carotenoid, has been shown to reduce the development of inflammatory polyarthritis in a population-based study [45]. Chlorogenic acid, the main bioactive phytochemicals in coffee, belongs to the polyphenol family and has been reported to have anti-gout activity, elicited via its anti-inflammatory effects [46]. Gout is a form of inflammatory arthritis that is highly associated with RA patients [47]. Triptolide shows inhibitory effects by decreasing IL-18 and IL-18 receptors in rheumatoid arthritis synovial fibroblasts stimulated by phorbol 12-myristate 13-acetate (PMA) [48]. Moreover, in an in vivo collagen-induced arthritis model, triptolide has been shown to exert immunosuppressive effects by decreasing the number of CD4+ T cells and increasing CD8+ T cells in Peyer’s patch from RA rats, resulting in the delay of arthritic progression [49] (Table 1).

**Table 1:** Phytochemicals and their effective mechanism in autoimmune diseases.

**SLE**							
**Class of Phytochemicals**	**Phytochemical Name**	**Experimental System**	**Effective Doses**	**Translated into Human-Relevant Equivalent**	**Mechanism of Actions**	**Main Source**	**Ref.**
Phenolic	Resveratrol 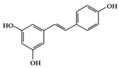	Lupus-prone mice	50 mg/kg	4 mg/kg	Reduction of B and Th1 cell percentage	Grapes, red wine	[25]
	Apigenin 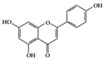	Lupus-prone mice	20 mg/kg	1.6 mg/kg	Suppression of IFN-γ, IL-17, and COX-2	Parsley, thyme, peppermint, olives, and chamomile	[26]
	Astilbin 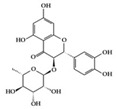	Lupus-prone mice	20 mg/kg	1.6 mg/kg	Reduction of IFN-γ, IL-17, TNF-α, IL-1β, and IL-6	*Smilax glabra*	[27]
	EGCG 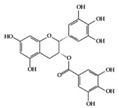	Lupus-prone mice	120 mg/kg	9.7 mg/kg	Reduction of NF-κB activation, NLRP3, IL-1β, and IL-18	Green tea	[28]
	Soy isoflavone (daidzin, glycitin, and genistin)	Lupus-prone mice	20 mg/kg	1.6 mg/kg	Reduction of IFN-γ	Soy food	[29]
**Rheumatoid Arthritis**							
**Class of Phytochemicals**	**Phytochemical Name**	**Experimental System**	**Effective Doses**	**Translated into Human-Relevant Equivalent**	**Mechanism of Actions**	**Main Source**	**Ref.**
Phenolics	Curcumin 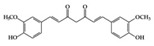	RA-induced rats	4 mg/mL (IV injection)	0.6 mg/mL	Reduction of TNF-α, IL-1β, and NF-κB activation	*Curcuma longa* Linn (turmeric)	[39]
	Resveratrol 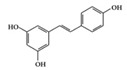	RA-induced rats	10 mg/kg	1.6 mg/kg	Reduction of TNF-α, CRP, and MPO Increase of IL-10	Grapes, red wine	[40]
	EGCG 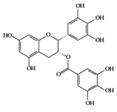	RA-induced mice	10 mg/kg	0.8 mg/kg	Reduction of TNF-α, IL-1β, IL-6, and IFN-γ	Green tea	[41]
	Mangiferin 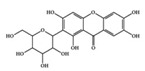	RA-induced mice	50, 100, 400 mg/kg	4, 8.1, 32.4 mg/kg	Reduction of TNF-α, IL-1β, IL-6, and RANKL	Thymelaeaceae family, ongael	[42]
	p-coumaric acid 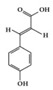	RA-induced rats	100 mg/kg	16.2 mg/kg	Reduction of TNF-α	Maize, wheat, apples, grapes, tomatoes, and spinach	[43]
	Genistein 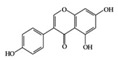	RA-induced rats	1 mL of 20 mg/mL/kg	3.2 mg/mL/kg	Reduction of IFN-γ and Increase of IL-4	Soy	[44]
	Chlorogenic acid 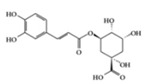	PO-induced mice, MSU-crystal induced rats	50, 100, 200 mg/kg (mouse) 40, 80, 160 mg/kg (rat)	4.0, 6.4, 8.1, 12.9, 16.2, 25.9 mg/kg	Reduction of TNF-α, IL-1β, IL-6	Chinese medicine	[46]
Terpenoid	Triptolide 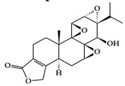	RA synovial fibroblast, RA-induced rats	100 ng/mL, 9.31, 18.62 µg/kg	1.51, 3.02 µg/kg	Reduction of IL-18 and IL-18 receptor	*Tripterygium wilfordii* Hook. f.	[48,49]

Currently, diverse antirheumatic drugs have been used in the treatment of RA, which is classified into two types; traditional disease-modifying antirheumatic drugs (DMARDs) and biologic DMARDs, and most of them have been known to have adverse effects [50,51]. Traditional DMARDs (methotrexate and sulfasalazine) are well known to have common side effects, including nausea and diarrhea. In particular, methotrexates are also known to have severe side effects such as pulmonary fibrosis [51]. Infliximab (anti-TNF-α antibody) as biologic DMARD has been reported to increase the risk of infection because of immune suppression [52]. In addition, it may provoke severe side effects such as anti-TNF-α-induced lupus or hepatitis, even though it is rare [53,54]. In Table 1, effective doses of phytochemicals in RA induced animal models are translated into human-relevant equivalents. Translated doses of anti-inflammatory phytochemicals showed to reduce RA severity ranged from 1.6 µg/kg to 32.4 mg/kg. Particularly, triptolide worked at 0.5 µg/kg of the lowest dose among several anti-inflammatory phytochemicals in RA-induced rats. The dose of 0.5 µg/kg is much lower than the effective dose (3 mg/kg) of infliximab [53,54]. Considering that current antirheumatic drugs have variable adverse effects, anti-inflammatory phytochemicals could be used as alternative medicines to treat RA [55].

### 2.2. Diabetes and Obesity

Diabetes is an increasingly common metabolic disease worldwide and is classified into two main types: type 1 diabetes mellitus (T1DM) and type 2 diabetes mellitus (T2DM) [56]. T1DM is a chronic inflammatory disease of pancreatic β-cell destruction that results in consistent pancreatic islet inflammation [57,58]. T2DM has shown that chronic inflammation is associated with pathogenesis through extensive research [59]. Obesity is also a global disease that involves chronic inflammatory processes in adipose tissue, even though it has many causative origins [60]. Additionally, many studies support that obesity and T2DM are closely linked [61]. Antidiabetic and anti-obesity drugs have been widely developed and used; however, they are not always very effective and often have undesired side effects. Therefore, natural bioactive compounds from plants have been extensively studied for their anti-inflammatory mechanisms against diabetes and obesity, and these plant-derived compounds could be alternatives to suppress and control diabetes and obesity without any adverse effects.

Many phenolic compounds have been characterized to have antidiabetic or anti-obesity effects on adipose tissues through different mechanisms [62,63]. Here, we mainly focused on the anti-inflammatory effects of phenolic compounds against diabetes and obesity (Table 2). Curcumin attenuates vascular inflammation in diabetes-induced rats by reducing leukocyte adhesion to the endothelium and decreasing ROS by downregulating elevated malondialdehyde (MDA) levels [64]. Another study showed that curcumin decreased IL-1β expression and NF-κB activation in the retina of diabetes-induced rats, suggesting that curcumin may have therapeutic potential against retinopathy in diabetic patients [65]. In addition, curcumin has beneficial effects on body weight by ameliorating adipose and hepatic inflammation in a high-fat diet (HFD)-induced obese mice [66]. Resveratrol attenuates vascular inflammation by decreasing the expression of Intercellular adhesion molecule 1 (ICAM-1), vascular cell adhesion protein 1 (VCAM-1), and monocyte chemoattractant protein 1 (MCP-1) and reduces macrophage infiltration in the aortic tissue of db/db mice a well-established model of obesity and diabetes [67]. Administration of resveratrol also reduces the percentage of induced cerebral infarction by decreasing TNF-α, IL-6, and MPO levels and increasing IL-10 in diabetic rats [68]. Quercetin shows anti-inflammatory effects by ameliorating serum TNF-α and CRP levels and inhibiting aortic NF-κB activation in both insulin-deficient and insulin-resistant rats [69]. Another study reported that quercetin has an anti-inflammatory effect by ameliorating TNF-α production in the adipose tissue of obese Zucker rats, resulting in a reduction in body weight [70]. Eriodictyol has anti-inflammatory effects on the retina of streptozotocin (STZ)-induced diabetic rats by decreasing the expression of TNF-α, ICAM-1, vascular endothelial growth factor (VEGF), and endothelial nitric oxide synthase (eNOS) proteins [71]. Naringenin, a common flavonoid found in many fruits, attenuates inflammatory cytokines (IL-1β, IL-6, and MCP-1) and suppresses fibrotic factors (fibronectin and transforming growth factor-β (TGF-β)) in the kidneys of diabetic mice, suggesting that naringenin may be helpful for diabetic nephropathy [72]. Another common flavonoid, hesperetin, reduces inflammatory cytokines (TNF-α and IL-1β) in diabetic rat retinas [73]. Baicalein, a flavone isolated from *Scutellaria baicalensis*, has preventive effects in a diabetic retinopathy rat model and acts by reducing IL-18, TNF-α, and IL-1β expression [74]. Another study has shown that baicalein exerts preventive effects on renal dysfunction in diabetic rats by ameliorating renal inflammatory processes by reducing the expression of NF-κB p65, inducible nitric oxide synthase (iNOS), and TGF-β in the kidney [75]. Baicalein treatment in HFD-induced obesity and diabetic mice was found to inhibit inflammation through the downregulation of the mitogen-activated protein kinase (MAPK) pathway [76]. Chrysin, an abundant flavone found in honey and plant extracts, acts as an anti-inflammatory compound and prevents renal dysfunction by suppressing renal TNF-α expression and NF-κB activation in the diabetic rat kidney [77]. Flavonol morin attenuates osteopenia in diabetic mice by decreasing the levels of pro-inflammatory cytokines, IL-6, TNF-α, and IL-1β [78]. Isoflavone genistein shows inhibitory effects on cardiac inflammation by reducing TNF-α, CRP, and TGF-β levels in the heart of diabetic rats [79]. Alkaloid colchicine ameliorates inflammatory cell infiltration by decreasing the expression of MCP-1 and ICAM-1 in the renal tissue of diabetic rats [80]. Another alkaloid capsaicin, a spicy component of hot pepper, improves obesity-related pathologies by inhibiting adipokines such as MCP-1 and IL-6 as well as by suppressing the migration of macrophages into adipose tissue in HFD-induced obese mice [81]. Recently, parthenolide, a feverfew-derived phytochemical, was reported to have anti-obesity effects (suppression of the increase in body weight) through the downregulation of inflammatory responses via inhibition of NF-κB and MAPKs in HFD-fed mice [82].

Many phytochemicals have anti-inflammatory effects by inhibiting inflammatory factors in db/db mice, STZ-induced diabetes rats, and HFD mice for T1DM, T2DM, and obesity disease models. Natural products for type 2 diabetes treatment have been known to have a variety of action mechanisms, including inhibition of glucose absorption (inhibition of α-glucosidase and α-amylase) in the digestive tract, increase in glucose uptake, effects on glucose transporters, enhancement of pancreatic β cell proliferation and insulin secretion, inhibition of protein tyrosine phosphatase 1B activity, and anti-inflammation [83,84]. Here, these phytochemicals ameliorate symptoms in diabetes-induced organ dysfunction such as retinopathy and nephropathy and improve insulin resistance. Moreover, some of these have been shown to have antidiabetic effects by mediating anti-inflammation. For example, naringenin alleviates the level of blood glucose by reducing inflammation in HFD and STZ-induced type 2 diabetes rats [85]. Vanillic acid has also been reported to decrease insulin resistance through its anti-inflammatory effects in HFD-induced rats [86]. Furthermore, kaempferol ameliorates insulin resistance by modulating inflammatory signaling in HFD and STZ-induced type 2 diabetic rats [87]. In addition, aggregation of islet amyloid polypeptide (IAPP) has been reported to impair β-cell insulin secretion by triggering the islet-mediated secretion of pro-inflammatory cytokines [88]. Several phytochemicals can block IAPP aggregation-related toxicity by preventing oligomer formation or inhibiting ROS and inflammatory processes induced by toxic oligomer formation [89]. Resveratrol has been reported to inhibit IAPP aggregation by integrating into the hydrophobic pocket between two IAPP molecules [90]. EGCG is known to disassemble IAPP-derived amyloid fibrils [91]. In addition, curcumin is thought to affect amyloid assemblies by preventing the helix-helix interaction of IAAP [92].

Taken together, we suggest that these anti-inflammatory phytochemicals could be direct candidates for developing antidiabetic drugs and could be a complementary and alternative medicine (CAM), even though the precise/effective dose and side effects need to be determined by clinical trials. In terms of the guidelines for diabetes treatment, metformin and miglitol have a dose range of 20–200 mg/kg and 10 mg/kg as the current drugs for treating human type II diabetes, respectively. In Table 2, effective doses of these phytochemicals in an animal model of type 2 diabetes are translated into human-relevant equivalents. Translated doses of anti-inflammatory phytochemicals that directly showed antidiabetic effects ranged from a minimum of 4.84 mg/kg to a maximum of 24.32 mg/kg (naringenin: 8.11–16.22 mg/kg, vanillic acid: 4.84 mg/kg, and kaempferol: 8.11–24.32 mg/kg). If these phytochemicals are applied using the guidelines for type 2 diabetes treatment, human-relevant equivalent doses of phytochemicals can be comparable with those of drugs for type 2 diabetes treatment. Furthermore, phytochemicals have been reported to have advantages with few side effects for type 2 diabetes treatment [84]. In addition, the use of some phytochemicals, such as curcumin, quercetin, capsaicin, and parthenolide, shows that the downregulation of inflammatory responses in adipose tissue is correlated with the reduction of body weight, which indicates that particular phytochemicals have the potential to act as functional agents against obesity. Even though obesity has a multifaceted etiology, low-grade inflammation commonly occurs in obesity [93]. In this respect, several phytochemicals modulating different inflammatory cascades can be exploited for adjunctive therapy for obesity. Given that there are several phytochemicals exhibiting different mechanisms of anti-inflammatory effects in both obese and diabetic model systems, combinatorial use of these compounds at lower doses could be applied for obesity as well as a diabetes treatment. Several anti-obesity drugs are known to have variable efficacy and side effects [94]. Phytochemicals with lower side effects than synthetic drugs could be an important source for CAM against diabetes and obesity [95,96].

**Table 2:** Phytochemicals and their effective mechanism in diabetes and obesity.

Class of Phytochemicals	Phytochemical Name	Experimental System	Effective Doses	Translated into Human-Relevant Equivalent	Mechanism of Actions	Main Source	Ref.
Phenolic	Curcumin 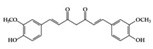	Diabetes-induced rats	300 mg/kg	48.6 mg/kg	Reduction of MDA level Inhibition of IL-1β and NF-κB activation	*Curcuma longa* Linn (turmeric)	[64,65]
	Resveratrol 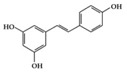	db/db mice, Diabetes-induced rats	20 mg/kg	1.6 mg/kg	Reduction of ICAM, VCAM, and MCP-1 Decrease of TNF-α, IL-6 and MPO level	Mulberries, peanuts, grapes, red wine	[67,68]
	Quercetin 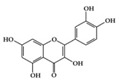	Diabetes-induced rats	50 mg/kg	3.2 mg/kg	Reduction of TNF-α and CRP	vegetables, fruits, leaves and seeds	[69]
	Eriodictyol 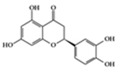	Diabetes-induced rats	10 mg/kg	1.6 mg/kg	Reduction TNF-α, ICAM-1, VEGF and eNOS	*Eriodictyon californicum*	[71]
	Naringenin 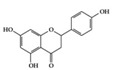	Diabetes-induced mice	2% of diet	0.16% of diet	Reduction of IL-1β, IL-6, MCP-1, and TGF- β	Grapefruit (*Citrus paradisi*), oranges (*Citrus sinensis*)	[72]
	Hesperetin 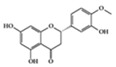	Diabetes-induced rats	100 mg/kg	16.2 mg/kg	Reduction of TNF-α and IL-1β	*Citrus* fruits	[73]
	Baicalein 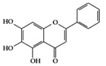	Diabetic retinopathy rat	150 mg/kg	24.3 mg/kg	Reduction of IL-18, TNF-α, and IL-1β	*Scutellaria baicalensis*, *Oroxylum indicum*	[74]
	Chrysin 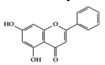	Diabetic nephropathy rat	40 mg/kg	6.4 mg/kg	Reduction of TNF-α and NF-κB activation	Many plant extracts, honey, and propolis	[77]
	Morin 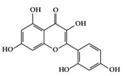	Diabetes-induced mice	30 mg/kg	2.4 mg/kg	Reduction of TNF-α, IL-1β, and IL-6	*Moraceae* family, weeds, mill, fig, almond, red wine and osage	[78]
	Genistein 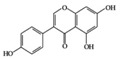	Diabetes-induced rats	300 mg/kg	48.6 mg/kg	Reduction of TNF-α, CRP, and TGF-β	Soybeans	[79]
	Vanillic acid 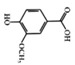	HFD-induced rats	30 mg/kg	4.84 mg/kg	Reduction of IL-1β, IL-6, MCP-1, and COX2	Vanilla beans	[86]
	Kaempferol 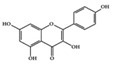	HFD and STZ-induced type 2 diabetic rats	50, 150 mg/kg	8.11, 24.32 mg/kg	Inhibition of IRS-1& IKKα/β phosphorylation and NF-κB activation	Broccoli, cabbage, kale, and beans	[87]
Terpenoid	Parthenolide 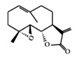	HFD-fed mice	1,10 mg/kg	0.08, 0.8 mg/kg	Downregulation of NF-κB and MAPKs Upregulation of Nrf2 and its target molecule, HO-1	Feverfew (*Tanacetum* *parthenium* (L.) Sch.Bip.)	[82]
Alkaloid	Colchicine 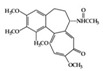	Diabetes-induced rats	30 µg/kg	4.8 µg/kg	Reduction of ICAM-1 and MCP-1	Autumn crocus *Colchicum autumnale*	[80]
	Capsaicin 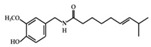	HFD-fed mice	2 mg/kg	0.14 mg/kg	Downregulation of IL-6, MCP-1, and NF-κB	Hot peppers	[81]

### 2.3. Neurodegenerative Diseases

Neurodegenerative diseases (ND) are age-related disorders, including Alzheimer′s (AD) and Parkinson’s disease (PD), whose pathogenesis includes various biological processes, oxidative damage, mitochondrial dysfunction, and chronic neuroinflammation [97]. Accumulating evidence has shown that neuroinflammation is closely related to ND pathogenesis. Recently, numerous plant compounds have been extensively studied to slow down at the initial stages and progression of ND. Several of these compounds have been found to control or suppress neuroinflammation. This section discusses the anti-inflammatory features of phytochemicals and their effect on the pathogenesis of ND.

Curcumin exhibits inhibitory effects on neuroinflammation in p25 overexpressing transgenic mice, which undergo neurodegenerative changes and vigorous neuroinflammation by downregulating pro-inflammatory cytokine expression (macrophage inflammatory protein-1α (MIP-1α), TNF-α, and IL-1β) and attenuating AD-like neuropathology [98]. Curcumin also exerts neuroprotective effects on lumbar radiculopathy by reducing COX-2 and IL-6 expression in TNF-α-induced dorsal root ganglion (DRG) neuroinflammation [99] and lipopolysaccharides (LPS)-induced PD model by significantly preventing pro-inflammatory cytokines (TNF-α, IL-1β, and IL-1α), iNOS, and NF-κB activation [100]. Caffeine is a well-known methylxanthine alkaloid isolated from the seeds of many plants, such as coffee beans. Caffeine treatment was found to attenuate pro-inflammatory markers (CCL4, TNF-α, and CCL5) in the hippocampus of AD mice with overexpressed mutated tau protein [101]. As shown in Table 3, caffeine can be translated into a human-relevant equivalent of 4.8 mg/kg (288 mg/day based on 60 kg adults), which is much less than 300–400 mg/day of caffeine for adults drinking modest amounts of coffee [102]. Acacetin has anti-neuroinflammatory effects in a 1-methyl-4-phenyl-1,2,3,6-tetrahydropyridine (MPTP)-induced PD mouse model by suppressing microglial cells, inhibiting iNOS and COX-2 expression, and reducing dopaminergic cell body loss [103] (Table 3).

The association between anti-inflammatory drugs such as nonsteroidal anti-inflammatory drugs (NSAIDs) and the occurrence of AD was studied, and the use of NSAIDs can prevent AD [104]. Currently, anti-AD drugs are not used to cure ND as much as to help mental function, although these drugs do not include anti-inflammatory drugs [105,106]. Moreover, AD is caused by the inflammatory response induced by the aggregation of the extracellular amyloid-β peptide (Aβ), which is closely linked to the pathogenesis of AD [107]. Therefore, one of the effective therapies for AD is the development of inhibitors of Aβ aggregation [108], and several reports have suggested the applicability of phytochemicals as Aβ aggregation inhibitors. EGCG has been reported to inhibit Aβ aggregation and Aβ42-induced ROS generation with a short fragment of Aβ [109]. Curcumin can also inhibit the self-aggregation of Aβ by binding to Aβ [110]. In addition, silybins have been reported to act as anti-aggregating compounds that inhibit the self-assembly of Aβ [111]. In this respect, phytochemicals with anti-neuroinflammatory effects could be a good source for developing new neuroprotective drugs that prevent or delay ND initiation and progression by inhibiting neuroinflammation.

## 3. Molecular Effects of Phytochemicals in Signaling Pathways Based on Inflammation-Associated Transcription Factors

**Figure 1:**
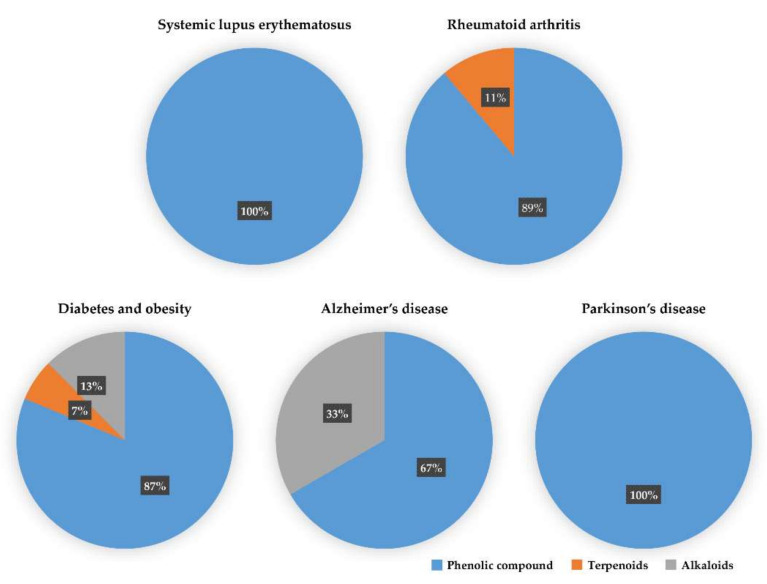
The percentage of effective phytochemicals in each prevalent inflammatory disease according to the summary of Table 1, Table 2 and Table 3. Phenolic compounds (blue) dominate all inflammatory diseases. Terpenoids (orange) are secondary dominant compounds in rheumatoid arthritis. In Alzheimer’s disease, alkaloids (gray) are secondary dominant compounds.

We reviewed a wide array of phytochemicals with anti-inflammatory effects on prevalent chronic inflammatory diseases in Section 2 and summarized the action mechanism and distribution between the different disease types and phytochemicals in Figure 1 and Table 1, Table 2 and Table 3. As shown in Figure 1, the distributions of phytochemicals accounted for the phenolics (89%) and terpenoids (11%) in the RA and phenolics (87%), terpenoids (7%), and alkaloids (13%) in diabetes/obesity. In addition, phenolics (67%) and alkaloids (33%) were identified in AD. All phytochemicals were phenolic in the PD and SLE groups. Regarding the relationship between the structures and anti-inflammatory activities of phytochemicals, approximately 80% of all anti-inflammatory phytochemicals that we reviewed belong to phenolics. Flavonoids are the most common among the phenolic subgroups (71% of phenolic phytochemicals). Flavone, flavanol, isoflavone, flavonol, and flavanone are almost evenly distributed among the flavonoids. We further analyzed the anti-inflammatory phytochemicals based on individual diseases. Flavonoids accounted for about 80%, 30%, and 75% of the compounds effective against SLE, RA, and diabetes/obesity, respectively. In this respect, it could be considered that the structure of the flavonoid backbone may be responsible for mediating these anti-inflammatory functions. Although the distribution shows that the proportion of polyphenols and flavonoids is more significant than that of other phytochemicals, each phytochemical exhibits anti-inflammatory effects in vivo through specific mechanisms. Each phytochemical belonging to the same class of phytochemicals exhibits a different action mechanism in individual chronic diseases. Furthermore, the same phytochemical may exhibit diverse anti-inflammatory effects in different types of inflammatory disease models. For instance, resveratrol ameliorated the disease symptoms by reducing B and Th1 cells in lupus-prone mice, decreasing inflammatory cytokines (TNF-α and IL-1β) in RA-induced rats and inhibiting cell adhesion molecules (ICAM and VCAM) in db/db mice. It appears that each phytochemical could have a cell type- or tissue type-specific mechanism in different cell/tissue types. In addition, any modification in the phytochemical structure is thought to influence its anti-inflammatory bioactivity [112]. Revealing the common principle of structure–activity relationships based on the backbone structure and backbone modification is a challenging issue. However, as the knowledge of structure–activity relationship is important for developing new anti-inflammatory agents, more in-depth research and high-throughput analysis of structure–activity relationships are required.

The regulation of both pro-inflammatory and anti-inflammatory cytokines is thought to be under inflammation-associated transcription factor signaling pathways (NF-κB, Janus kinase (JAK)-Signal transducer and activator of transcription (STAT), and nuclear factor erythroid 2 related factor 2 (Nrf2)) in chronic inflammatory diseases. The related genetic regulation resulting in pro- or anti-inflammatory mediator secretion is generally dependent on the activation of NF-κB transcription [113], leading to crosstalk with other transcription signaling pathways by inflammatory stimuli [114,115,116]. As shown in Table 1, Table 2 and Table 3, most phytochemical action mechanisms account for the NF-κB signaling pathway, and minor phytochemicals use other pathways. In particular, the downregulation of iNOS and COX-2, two necessary inducible enzymes that play a critical role in NO and prostaglandin E2 (PGE2) production, are well known to underlie the NF-κB signaling pathway [117]. Additionally, many previous studies have demonstrated that the Nrf2 signaling pathway is also involved in anti-inflammatory processes by regulating various inflammatory mediators [114]. Several phytochemicals in Section 2 are against inflammation by directly increasing Nrf2 activation or inducing antioxidant enzymes in pathological inflammatory processes. Here, the intracellular inflammatory signaling pathway modulated by phytochemicals listed in Table 1, Table 2 and Table 3 is presented by focusing on inflammation-associated transcription factors in the following text, which should help understand the therapeutic effects of numerous anti-inflammatory phytochemicals in chronic inflammatory diseases. In addition, we further analyzed whether each phytochemical is linked to the type of transcription factor, in Table 4. As shown in Table 4, a large portion of phytochemicals acting on NF-κB signaling pathways belongs to the class of flavonoids (70%). It seems that the backbone of flavonoids may be important for mediating these anti-inflammatory functions through the regulation of NF-κB signaling pathways. On the other hand, many phytochemicals can be categorized into different groups or subgroups based on their structures. Diverse phytochemicals belonging to different structural groups have anti-inflammatory effects on the same transcription factor pathways (Table 4). In addition to the backbones, differently modified functional groups of phytochemicals should be considered for the analysis of structure-based activity relationships. Therefore, in-depth studies should delineate the relationship between the structures and the anti-inflammatory activities of phytochemicals based on a particular step of the inflammatory signaling cascades.

**Figure 2:**
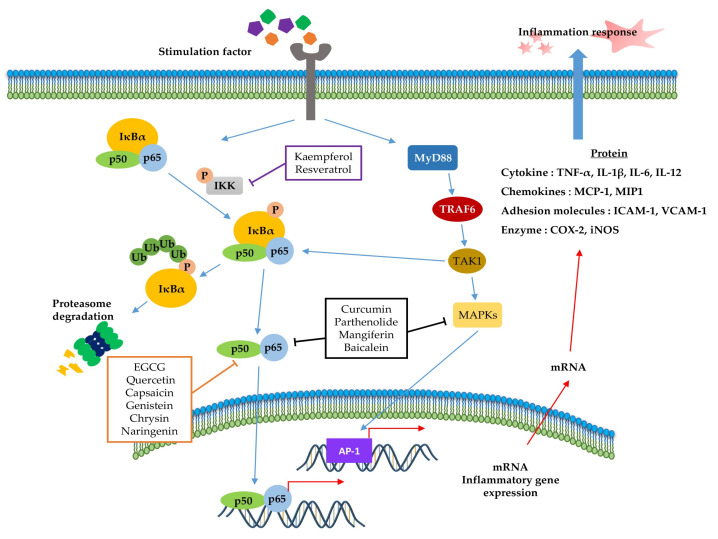
Role of the inflammation-associated transcription factor signaling pathway (NF-κB) and mitogen-activated protein kinase (MAPK) pathways in the inflammatory response. NF-κB and MAPK signaling can be activated by the binding of diverse ligands to receptors. In NF-κB signaling, various adapter proteins and signaling kinases lead to activation of the IKK complex, which then phosphorylates IκBα. Proteasomes can degrade phosphorylated IκBα. The free p50/p65 complex can translocate to the nucleus and activate transcription of active genes, such as cytokines, chemokines, adhesion molecules, and enzyme-related pro-inflammatory factors. In MAPK signaling, activated Myd88 by stimuli recruits TRAF6 to assemble a MyD88 signaling complex and subsequently facilitates TAK1 activation by TRAF6. Activated TAK1 mediates the stimulation of MAPKs, which activate AP-1, a transcription factor involved in inflammation. Additionally, activator protein-1 (AP-1) and NF-κB can induce the transcription of inflammatory genes. The signaling steps are affected by phytochemicals in the NF-κB and MAPK pathway axes.

NF-κB (Figure 2) is a crucial regulator of inflammatory responses in both innate and adaptive immunity. It coordinates the expression of a variety of genes to elicit an appropriate inflammatory response [118]. The NF-κB family consists of five members (p50/p105, p52/p100, p65 (RelA), RelB, and C-Rel), which form homodimers or heterodimers with each other [119]. Under normal conditions, NF-κB is inactive in the cytosol by inhibitory kappa B (IκB), a major inhibitor of NF-κB. However, inflammatory inducers such as LPS cause phosphorylation of IκB by IκB kinase β (IKKβ), resulting in the dissociation of IκB from NF-κB and subsequent degradation of IκB via the ubiquitin-proteasome pathway [118,120,121]. Consequently, NF-κB is exposed and stimulated, allowing it to mediate the production of specific cytokines (TNF-α, IL-1, IL2, IL-6, and IL-8) as well as the expression of COX-2 and iNOS, which are inflammatory mediators [122]. Thus, inhibition of NF-κB can be a potential application in chronic inflammatory disease treatment [123]. Among the phytochemicals listed in Table 1, a variety of them inhibit NF-κB activation and reduce pathological inflammatory conditions in an in vivo disease model, which results in the attenuation of disease symptoms. For example, resveratrol [67] and kaempferol [87] inhibits the activation of NF-κB by downregulating IKK phosphorylation, resulting in the suppression of inflammatory cytokines, such as TNF-α, IL-12, and IL-6. In terms of interrelation between NF-κB and other pathways, it is identified that several phytochemicals (Table 1, Table 2 and Table 3) improve inflammatory disorder symptoms by inhibiting NF-κB activation together with the suppression of other transcription signal pathways. In addition, MAPKs are a family of serine/threonine protein kinases, including ERK1/2, c-Jun N-terminal kinase (JNK), and p38 MAPK, which regulate gene transcription involved in inflammation [117]. JNK and p38 are activated by inflammatory stimuli and stress, whereas ERKs are usually activated by mitogens and differentiation signals [124,125]. Activation of MAPKs transforms extracellular stimuli such as oxidative and pro-inflammatory stimuli into the activation of transcription factors (activator protein-1 (AP-1): Fos + Jun), which regulates the production of NO and induces the upregulation of inflammatory gene expression [126]. Many previous studies have reported that MAPKs crosstalk with other pathways, such as NF-κB [127,128]. In particular, activation of NF-κB and MAPKs shares tumor necrosis factor receptor (TNFR)-associated factors (TRAFs) as upstream signaling events (Figure 2) [124]. Parthenolide [82] decrease the expression of inflammatory cytokines by reducing the phosphorylation of MAPK (ERK, JNK, and p38) and NF-κB. In addition, curcumin [99] and mangiferin [42] improve the inflammation reaction by inhibiting ERK activation, whereas baicalein [76] is dependent on the downregulation of ERK and JNK signaling. Taken together, the phytochemicals in this pathway commonly show anti-inflammatory effects by inhibiting NF-κB signaling and/or the MAPK signaling pathway.

**Figure 3:**
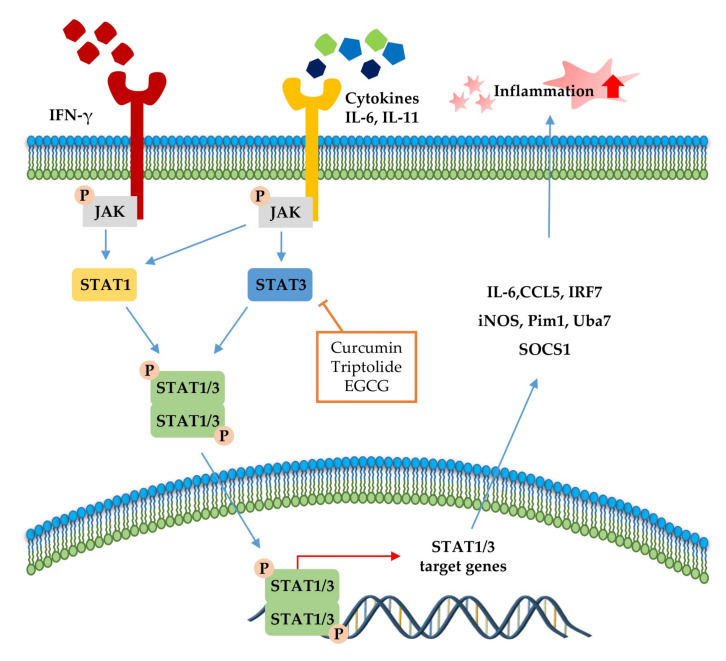
Signal transducer and activator of transcription (STAT)1 and STAT3 pathways in the inflammatory response. STAT signaling is activated by cytokines (IL-6, IL-11, and IFN-γ). Canonically, ligand binding to receptors triggers Janus kinase (JAK) phosphorylation and, subsequently, STATs, followed by STAT dimerization. The dimers translocate to the nucleus, where it drives transcription of target genes involved in inflammation. The signaling steps that are affected by phytochemicals in the STAT1/3 pathway axis are presented.

The JAK-STAT pathway (Figure 3) is a highly conserved signaling pathway capable of allowing extracellular factors to control gene expression [129]. Receptor-associated JAKs are activated when extracellular ligands bind to cytokine receptors and phosphorylate cytoplasmic transcription factor STATs [116]. In turn, STAT dimers translocate into the nucleus and bind to the target gene, resulting in the regulation of inflammatory gene expression [130]. In addition, STAT1 and STAT3 were reported to be closely associated with chronic inflammation [131,132]. As summarized in Table 4 and Figure 3, several phytochemicals (curcumin [133], triptolide [134], and EGCG [135]) ameliorate disease symptoms by inhibiting STAT3 in inflammatory disease model systems.

**Figure 4:**
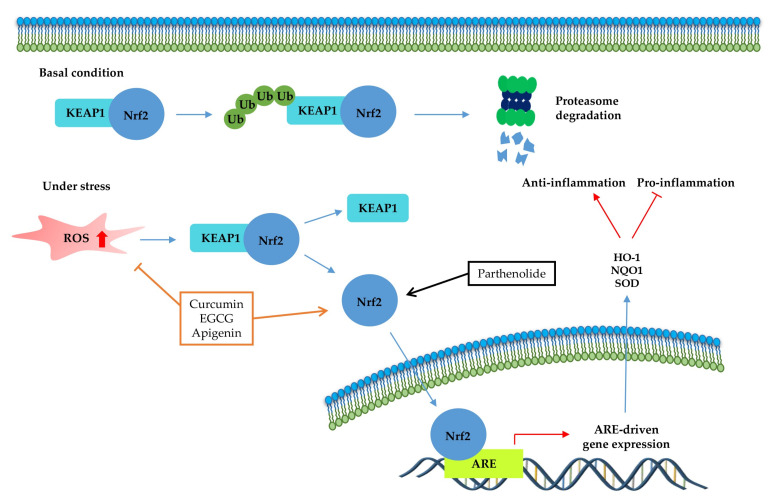
Kelch-like ECH-associated protein 1 (Nrf2-Keap1)-antioxidant response element (ARE) pathway. Under basal conditions, the Nrf2/Keap1 complex is a prerequisite for its subsequent polyubiquitination, resulting in proteasomal degradation of this complex. Under oxidative stress, free Nrf2 can translocate to the nucleus and activate ARE-related gene transcription. The signaling steps that are affected by phytochemicals in the Nrf2-signaling axis are presented.

The transcription factor Nrf2 (Figure 4) is the primary factor involved in the inducible cell defense system, regulating the expression of oxidative stress-related genes [136]. The target of Nrf-2 is the antioxidant response element (ARE) in the promoter regulatory regions of cytoprotective genes or antioxidant enzymes, including NAD(P)H quinone oxidoreductase 1 (NQO1), heme oxygenase 1 (HO-1), and superoxide dismutase (SOD) [114,137]. The activation of Nrf2 is tightly regulated by Kelch-like ECH-associated protein 1 (Keap1), which mediates ubiquitin-dependent proteasomal degradation of Nrf2. Upon oxidative stress, the ubiquitination of Nrf2 is stopped by the inactivation of Keap1. As a result, the activated Nrf2 is translocated into the nucleus and induces the defensive system’s activation through modulation of the Keap1–Nrf2–ARE signaling pathway, regulating the redox state of cells to maintain intracellular homeostasis [114,136,138]. As shown in Table 4 and Figure 4, a couple of phytochemicals ameliorate the inflammatory response by directly upregulating Nrf2 activation. Curcumin [133], parthenolide [82] and EGCG [28,41] improved the pathological inflammatory condition in a mouse model by activating Nrf2, resulting in increased expression of downstream targets such as HO1, NQO1, or SOD. In addition, curcumin [139], EGCG [140], and apigenin [141] mitigate the disease symptoms by directly blocking ROS. These phytochemicals can have anti-inflammatory effects by acting as direct antioxidants and activating Nrf2 in redox-sensitive signaling.

**Figure 5:**
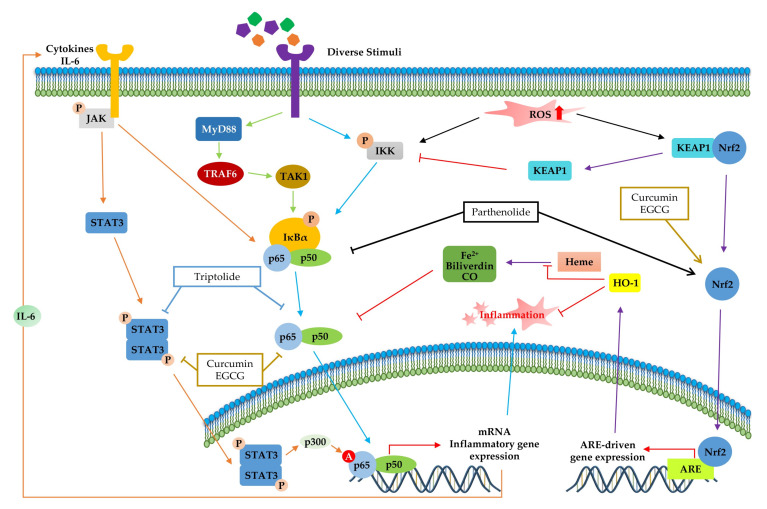
Crosstalk among inflammation-related transcription factors. The products of inflammatory mediators, such as cytokines and reactive oxygen species (ROS), are produced continuously by various inflammatory responses. Activated JAK and TAK1 modulate the phosphorylation of IκBα. Furthermore, the activated SATA3 dimer can translocate to the nucleus and activates the NF-κB transcription factor via p300-mediated acetylation. Accumulated ROS disrupts the Keap1-Nrf2 complex and upregulates the expression of ARE genes, such as heme oxygenase 1 (HO-1), by translocation of free nuclear factor erythroid 2 related factor 2 (Nrf2). HO-1 catalyzes heme and directly inhibits inflammation. The signaling steps that are affected by phytochemicals in multiple signaling pathways are presented.

Accumulating evidence has shown the crosstalk between NF-κB and other transcription factors in inflammation (Figure 5). Although dysregulation of inflammation-associated transcription factor signaling pathways (NF-κB, STAT, and Nrf-2) is most notably associated with chronic inflammatory diseases, phytochemicals can suppress inflammatory diseases by simultaneously regulating multiple inflammatory signaling pathways. In particular, IL-6 family members, which are the most notable inflammatory factors encoded by the NF-κB target gene, activated STAT3 protein [142,143]. Given the interrelation between STAT and NF-κB, several phytochemicals (curcumin, triptolide, and EGCG) improve inflammatory conditions by inhibiting both STAT3 and NF-κB activation, as shown in Figure 5. Moreover, the activation of NF-κB by sustained oxidative stress influences the Keap1–Nrf2–ARE signaling pathway [114]. The Keap1-Nrf2 complex is disrupted by ROS accumulation via an inflammatory response, and Keap1 inhibits IKK, which induces NF-κB activation [144]. Furthermore, the upregulation of ARE genes, such as HO-1, leads to translocation of free Nrf2 and increased ROS-catalyzed degradation of heme into bilirubin, free iron, and CO, which inhibits NF-κB [145]. Moreover, HO-1 directly regulates inflammation by inhibiting pro-inflammatory cytokines or activating anti-inflammatory cytokines [114]. As shown in Figure 5, parthenolide [82] modulates inflammatory signaling by inhibition of NF-κB and Nrf-2 pathways. In addition, curcumin [99,133] and EGCG [28,135] have anti-inflammatory effects by modulating all of inflammation-associated transcription factor pathways: NF-κB, STAT3 and Nrf2.

Therefore, these results suggest that various phytochemicals have anti-inflammatory effects by inhibiting pro-inflammatory mediators or inducing anti-inflammatory mediators via multiple regulations of the inflammatory transcription factor signaling pathway in chronic inflammatory diseases [146].

## 4. Conclusions and Perspectives

A growing body of evidence has shown that various phytochemicals have anti-inflammatory effects on various chronic inflammatory disorders [20]. They are characterized by increased expression of inflammatory cytokines, increased immune cell infiltration, and elevated ROS levels, which are implicated in the pathological conditions of various inflammatory diseases [18]. Here, we focused on phytochemicals that relieve disease symptoms by inhibiting inflammatory mediators in in vivo animal model systems and analyzed the distribution of classified phytochemicals in the chronic inflammation disease model system (as shown in Table 1, Table 2 and Table 3 and Figure 1). In addition, we showed that effective doses of these phytochemicals in animal model systems are translated into human-relevant equivalents, compared to current drugs used in chronic inflammatory diseases. Lastly, we analyzed the in-depth intracellular anti-inflammatory mechanisms of phytochemicals based on the inflammatory transcription factor-associated pathway in Section 3 and summarized them in Table 4. Many anti-inflammatory drugs have been developed and used against many inflammatory diseases, including AD, SLE, RA, and diabetics; however, long treatment durations often have undesirable side effects and are not always successful [22,147,148]. As mentioned in Section 2, phytochemicals could have advantages with no toxic or fewer side effects compared to synthetic chemical drugs that are currently used for the treatment of diabetes, SLE, and RA. In this regard, anti-inflammatory phytochemicals are potential therapeutic candidates for the development of drugs for inflammatory diseases. A myriad of phytochemicals has been studied for their roles in influencing anti-inflammatory mechanisms to prevent inflammation-associated disorders [149]. Understanding how phytochemicals can modulate intracellular signaling is very important for identifying specific molecular targets, which is essential for their use as therapeutic agents. In this respect, this review attempted to discuss anti-inflammatory phytochemicals in different chronic inflammatory diseases and convert the effective doses of phytochemicals in animal disease models into human-relevant equivalents (Table 1, Table 2 and Table 3 and Figure 1). When the effective doses of phytochemicals in mice or rat disease models are translated into the human-relevant equivalent, as shown in Table 1, Table 2 and Table 3, several phytochemicals show anti-inflammatory effects at a much lower dose than current anti-inflammatory drug doses used in different types of chronic inflammatory diseases. Even though phytochemicals could be a CAM, the precise/effective dose and a toxic dose of these anti-inflammatory phytochemicals need to be determined by clinical trials.

We summarized the molecular signaling mechanisms altered by phytochemicals in chronic inflammatory disease (Table 4), focusing on the inflammation-associated transcription factor, NF-κB (Figure 2), STAT (Figure 3), and Nrf-2 signaling pathways (Figure 4), which are the main potential anti-inflammatory targets, and crosstalk of inflammation-related transcription factor signaling pathways (Figure 5). The data we have summarized indicate that numerous plant-derived active compounds have been implicated in preventing specific molecular targets of inflammatory signaling cascades by reducing the levels of pro-inflammatory cytokines as well as COX-2, iNOS, and ROS, in in vivo disease model systems. These biologically active phytochemicals show potential as novel anti-inflammatory drugs with few side effects in treating inflammation-related disorders. Future research aimed at accruing profound clinical evidence is pertinent. In addition, in the current coronavirus disease 2019 (COVID-19) pandemic situation, with no treatment and prevention strategies against severe acute respiratory syndrome coronavirus 2 (SARS-CoV2), phytochemicals may be considered as potential antiviral agents [150]. Accumulating evidence suggests that a variety of plant extracts and active phytochemicals exhibit antiviral activities against different viruses [151]. It has been reported that prevalent comorbidities such as diabetes or neurodegenerative diseases (AD and PD) worsen SARS-CoV2 disease outcomes [152,153]. Both diabetes and AD are characterized by chronic inflammatory conditions, which have been associated with worse outcomes in COVID-19 patients [152,154]. As anti-inflammatory phytochemicals can improve inflammatory disease condition and exert direct antiviral activities, they could be potential drug candidates against many viruses, including SARS-CoV2 [155,156]. The present review provides comprehensive information on numerous bioactive phytochemicals with established anti-inflammatory properties and specific molecular targets in animal models of various chronic inflammatory diseases. It also suggests the need for anti-inflammatory phytochemicals to develop drugs against inflammatory diseases in the presence of viral infections as well as against chronic inflammatory diseases.

## Figures and Tables

**Table 3:** Phytochemicals and their effective mechanism in neurodegenerative diseases.

**Alzheimer’s Disease**							
**Class of Phytochemicals**	**Phytochemical Name**	**Experimental System**	**Effective Doses**	**Translated into Human-Relevant Equivalent**	**Mechanism of Actions**	**Main Source**	**Ref.**
Phenolic	Curcumin 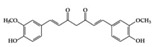	p25 overexpressing mice, TNF-α-induced DRG neurons	800 mg/kg	64.8 mg/kg	Reduction of TNF-α, IL-1β, and MIP-1α Reduction of COX-2 and IL-6	*Curcuma longa* Linn (turmeric)	[98,99]
Alkaloid	Caffeine 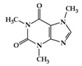	Tau overexpressed AD mice	0.3 g/L	0.02 g/L	Reduction of CCL4, CCL5, and TNF-α	Coffee	[101]
**Parkinson’s Disease**							
**Class of Phytochemicals**	**Phytochemical Name**	**Experimental System**	**Effective Doses**	**Translated into Human-Relevant Equivalent**	**Mechanism of Actions**	**Main Source**	**Ref.**
Phenolic	Curcumin 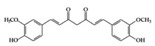	LPS-induced PD rats, TNF-α-induced DRG neurons	40 mg/kg	6.4 mg/kg	Reduction of TNF-α, IL-1β, IL-1α, iNOS, and NF-κB activation	*Curcuma longa* Linn (turmeric)	[100]
	Acacetin 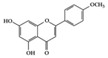	MPTP-induced PD mice	10 mg/kg	0.8 mg/kg	Inhibition of iNOS and COX-2	*R. pseudoacacia,* Fabaceae family	[103]

**Table 4:** Phytochemical categorization depending on the inflammation-associated transcription factor.

Transcription Factor	Phytochemical		
	Phenolics	Terpenoids	Alkaloids
**NF-κB**	Phenolic acid: curcumin, Stilbenes: resveratrol Flavones: chrysin, baicalein Flavanols: EGCG Flavonols: quercetin, kaempferol Flavanone: naringenin Isoflavones: genistein Xanthonoid: mangiferin	Sesquiterpene: parthenolide	Protoalkaloid: capsaicin
**JAK-STAT**	Phenolic acid: curcumin Flavanols: EGCG	Diterpenoid: triptolide	
**Nrf2**	Phenolic acid: curcumin Flavanols: EGCG Flavones: apigenin	Sesquiterpene: parthenolide	
